# Effects of ocean warming and coral bleaching on aerosol emissions in the Great Barrier Reef, Australia

**DOI:** 10.1038/s41598-018-32470-7

**Published:** 2018-09-19

**Authors:** Rebecca Jackson, Albert Gabric, Roger Cropp

**Affiliations:** 1 School of Environment and Science, Griffith University, Gold Coast, 4222 Australia; 2 Australian Rivers Institute, Griffith University, Gold Coast, 4222 Australia; 3 School of Environment and Science, Griffith University, Nathan, 4111 Australia

## Abstract

It is proposed that emissions of volatile sulfur compounds by coral reefs contribute to the formation of a biologically-derived feedback on sea surface temperature (SST) through the formation of marine biogenic aerosol (MBA). The direction and strength of this feedback remains uncertain and constitutes a fundamental constraint on predicting the ability of corals to cope with future ocean warming. We investigate the effects of elevated SST and irradiance on satellite-derived fine-mode aerosol optical depth (AOD) throughout the Great Barrier Reef, Australia (GBR) over an 18-year time period. AOD is positively correlated with SST and irradiance and increases two-fold during spring and summer with high frequency variability. As the influence of non-biogenic and distant aerosol sources are found to be negligible, the results support recent findings that the 2,300 km stretch of coral reefs can be a substantial source of biogenic aerosol and thus, influence local ocean albedo. Importantly however, a tipping point in the coral stress response is identified, whereby thermal stress reaches a point that exceeds the capacity of corals to influence local atmospheric properties. Beyond this point, corals may become more susceptible to permanent damage with increasing stress, with potential implications for mass coral bleaching events.

## Introduction

Ocean warming poses one of the greatest threats to coral reefs worldwide, with the incidence of coral bleaching and mortality increasing as sea surface temperature (SST) continues to rise^1–4^. This is of great concern as coral reefs provide numerous essential ecosystem, economic and social services^5–7^. Evidence also suggests that coral reefs play an important role in local climate regulation through the production of marine biogenic aerosol (MBA)^8–10^. MBA is thought to influence the Earth’s radiative budget both directly through backscattering of incoming short-wave solar radiation, and indirectly through effects on cloud microphysics and cover, possibly mitigating some of the warming effects of greenhouse gases (GHG)^11^. The direction and strength of this feedback, and the extent to which coral reefs contribute to the aerosol burden remains uncertain and constitutes a fundamental constraint on our ability to accurately project future climate.

In the relatively unpolluted marine boundary layer (MBL) of the southern hemisphere, biogenic sulfur emissions constitute a major source of secondary MBA^12–14^. Coral reefs are recognised as being among the strongest sources of this natural sulfate through the production of dimethylsulfoniopropionate (DMSP), particularly during periods of physiological stress^8,15–18^. DMSP acts as a biologically important antioxidant^19–21^, cryoprotectant^22^, anti-grazing agent^23,24^, metabolic precursor^25^, chemo-attractant^26^ and osmoregulator^20^. DMSP is also the precursor to dimethylsulfide (DMS), a volatile gas which is oxidised by hydroxyl radicals (OH) in the atmosphere to sulfur dioxide (SO_2_), methane sulfonic acid (MSA) and sulfuric acid (H_2_SO_4_)^27^. These aerosol precursor compounds may contribute to the growth of existing particles or nucleate to form new non-sea salt sulfate (nss-SO_4_) particles which can act as efficient cloud condensation nuclei (CCN).

The CLAW hypothesis, proposed in 1987, suggested that enhanced DMS emissions led to an increase in cloud droplet number, a decrease in droplet size and thus, increased low-level cloud (LLC) cover, lifetime and albedo, acting to locally reduce SST through a biologically-derived negative feedback^11^. Yet despite decades of research, our knowledge of the complex nature of this feedback remains incomplete. Some researchers suggest that the original hypothesis is an over-simplification^28^ or no longer relevant with anthropogenic perturbation of atmospheric properties throughout much of the globe^29^. However, others remain steadfastly positive about the role of DMS in global climate^30^.

In pristine coral reefs such as the Western Pacific Warm Pool (WPWP), DMS emissions and enhanced LLC formation are thought to be the key drivers behind an ocean thermostat which suppresses ocean warming below coral thermal tolerance thresholds (~30 °C)^31–33^. Despite corals in this region living close to their thermal maxima, few coral bleaching events have been recorded over the past 25 years^31^. Evidence suggests that a similar mechanism exists in the Great Barrier Reef, Australia (GBR), where although ocean temperatures in north-eastern Australia are warming, SST in the northern GBR is rising at a slower rate compared to southern regions^34,35^. As in the WPWP, this may be due to the high biomass of DMS producing corals and the accumulation of DMS-rich air in the prevailing south-east trade winds over the GBR^10,16,36^. *Acropora* corals, the dominant genus throughout the GBR, are among the highest producers of DMS and enhance emissions in response to thermal, irradiance and osmotic stress^9,15,16,20,37^. Seasonal increases in DMS emissions from the GBR have therefore been observed during spring and summer, particularly during low tides and periods of high rainfall when aerial exposure and hypo-salinity affect coral physiology^15,38^.

The atmospheric residence time of DMS is approximately one day^39^, meaning that radiative effects of DMS-derived sulfate aerosols are often far from the source location. However, a strong link has been established between coral physiological stress and satellite-derived fine-mode aerosol over the southern GBR during calm conditions, when the advection of atmospheric particles and the influence of non-biogenic sources are minimal^8^. Strong nucleation events have also been observed over the GBR from compounds originating from the coral reef^12^. These findings support the conjecture that corals have the ability to influence local ocean albedo and thus, mitigate thermal stress.

Disturbingly however, mass coral bleaching events are occurring more frequently throughout the GBR, indicating that ongoing ocean warming is exceeding the capacity of corals natural protective mechanisms. Recent evidence shows that corals reduce emissions of aerosol precursor gases when SST and irradiance levels exceed their physiological limits. During these conditions endosymbiont photosystems produce excess amounts of harmful reactive oxygen species (ROS), causing an upregulation of the coral antioxidant response whereby the rate of intracellular DMSP consumption exceeds that of DMSP breakdown to DMS^9,40^. Although the extent to which coral reef DMS emissions contribute to aerosol formation is uncertain, reductions have the potential to cause a decline in CCN and LLC formation and an increase in irradiance, exacerbating coral stress and resulting in a positive feedback on SST. This raises concern as to whether corals in the GBR will be able to cope with further SST rise and suggests a potential weakening of any DMS-SST feedback mechanism.

Our current understanding of aerosol-climate interactions is limited and constitutes a fundamental constraint on predicting future climate scenarios^13,41^. This is particularly true of coral reef ecosystems where the relationship between coral physiological stress, changes to local atmospheric properties and the effect on regional climate is complex and poorly understood^10,28^. Our aim in this analysis is to investigate the relationship between satellite-derived SST, coral irradiance stress (IR) and fine-mode aerosol optical depth (AOD) over the GBR. Data on key oceanic and atmospheric parameters are used to examine how this relationship varies seasonally and with latitude from 2000 to 2017, a period that includes several mass coral bleaching events.

## Methods

### Study location

The GBR spans 2,300 km of the north-east Queensland (QLD) coastline between 10°S and 23°S, and encompasses an area of 347,000 km^2^, making it the largest living structure on the planet. The climate in north-eastern Australia ranges from sub-equatorial in the north, to sub-tropical in the south and is characterised by wet, hot summers (November to April) and dry, mild winters (May to October)^42^. The Great Barrier Reef Marine Park (GBRMP) was established by the Australian Government in 1975 and consists of four major management zones encompassing the southern, central, northern and far northern GBR. This analysis used four sites spanning the length of the GBRMP and representative of each management zone (Supplementary Fig. S1). These sites consisted of large grids of 1° × 1° to allow for the inclusion of satellite-derived parameters with varying spatial resolution up to 1°.

### Analysis

Area-averaged time series for key oceanic and atmospheric parameters were obtained for 2000 to 2017. Daily fine-mode (0.1–0.25 μm) AOD at 869 nm was obtained from NASA’s Level 1 Atmospheric Archive and Distribution System Distributed Active Archive Center (LAADS DAAC) (https://ladsweb.modaps.eosdis.nasa.gov/). This time-series was acquired from the Moderate Resolution Imaging Spectroradiometer (MODIS) sensor on board Terra and Aqua satellites which include daily morning and afternoon overpasses. Data on 8-day chlorophyll-a (as a proxy for phytoplankton biomass: CHL (mg m^−3^)), photosynthetically available radiation (PAR: einstein m^−2^) and diffuse water column attenuation coefficient (k490: m^−1^), acquired from MODIS Aqua, were obtained from the Goddard Earth Sciences Data Information Services Centre’s (GES DISC) Giovanni v4.24 online data system^43^. High-resolution blended analysis of daily SST (°C) was downloaded from the NOAA Earth System Research Laboratory Physical Sciences Division (https://www.esril.noaa.gov/psd/). Daily surface wind speed (WS: ms^−1^) and direction was obtained from NOAA Blended Ocean Winds Daily Aggregation (https://www.ncei.noaa.gov/thredds/blended-global/oceanWinds.html). Daily tide predictions were obtained from the Australian Government Bureau of Meteorology and used to calculate daily noon tide height (NT: m).

A measure of midday coral irradiance stress (IR) was calculated as a function of PAR, water clarity (k490) and NT similarly to Cropp *et al*. (2018) to examine the effects of elevated irradiance on AOD. PAR and k490 were obtained from MODIS Aqua which passes over the equator at 13:30 travelling north and therefore provides measures for both parameters over the GBR at approximately noon local time. The IR metric was greatest when PAR was high and both NT and k490 were low and therefore represents conditions likely to cause a stress response in corals. Exploratory analysis examined seasonal and latitudinal variability between AOD and each of the aforementioned parameters over the 18-year study period. Spearman’s ranked correlation analysis (α = 0.001) was used to account for non-normal distribution and the presence of outliers common in environmental data. Observations for all parameters were available on only 66% of the 6570-day study period and so 8-day means were used to allow for maximum data inclusion (southern GBR *n* = 690; central GBR n = 682; northern GBR n = 667; far northern GBR n = 658). Additionally, 8-day data is appropriate to account for the delay between DMS emission and subsequent oxidation and nucleation to form new particles^39^.

Short-term, high frequency (30-minute) field measurements of atmospheric DMS at Heron Island (southern GBR) were available from 5^th^ to 18^th^ February 2016 AEST^44^. These were averaged into hourly values and correlated with hourly mean SST (*n* = 274) obtained from the Australian Institute of Marine Science (AIMS) Integrated Marine Observing System (IMOS) Sensor Float 1^45^, located at 0.3 m depth in the Heron Island coral reef flat. DMS was not correlated with AOD or other parameters, as these data were only available on approximately nine days of the DMS sampling period.

Further analysis then examined the variability between AOD anomalies and SST during critical time periods surrounding mass coral bleaching events (southern GBR *n* = 833; central GBR *n* = 833; northern GBR *n* = 829; far northern GBR *n* = 824). Coral bleaching degree heating weeks (DHW: °C-weeks) were included as a measure of coral thermal stress intensity and duration to examine the effect of accumulated coral thermal stress on AOD^46–49^. Corals are considered to be stressed when SST exceeds the climatological mean monthly maximum (MMM)^47^. DHW was therefore calculated as a moving sum of the positive differences between the daily observed SST and the MMM over the most recent 84-day period, divided by seven to obtain a weekly value. As this measure is an accumulation of SST anomalies, a positive DHW value may occur when SST is not elevated, reflecting some exposure of corals to temperature stress over the past 12-week period. A value of 2 °C-weeks may therefore reflect one week of SST 2 °C above the MMM or two weeks of SST 1 °C above the MMM, with both conditions theoretically eliciting similar stress levels in coral. In several remote sensing studies, 4 °C-weeks indicate accumulated thermal stress sufficient to cause coral bleaching, while a value of 8 °C-weeks or higher indicates extreme warming, likely to result in mass coral bleaching and mortality^46,47^.

## Results

### Seasonal and latitudinal variability

**Figure 1:**
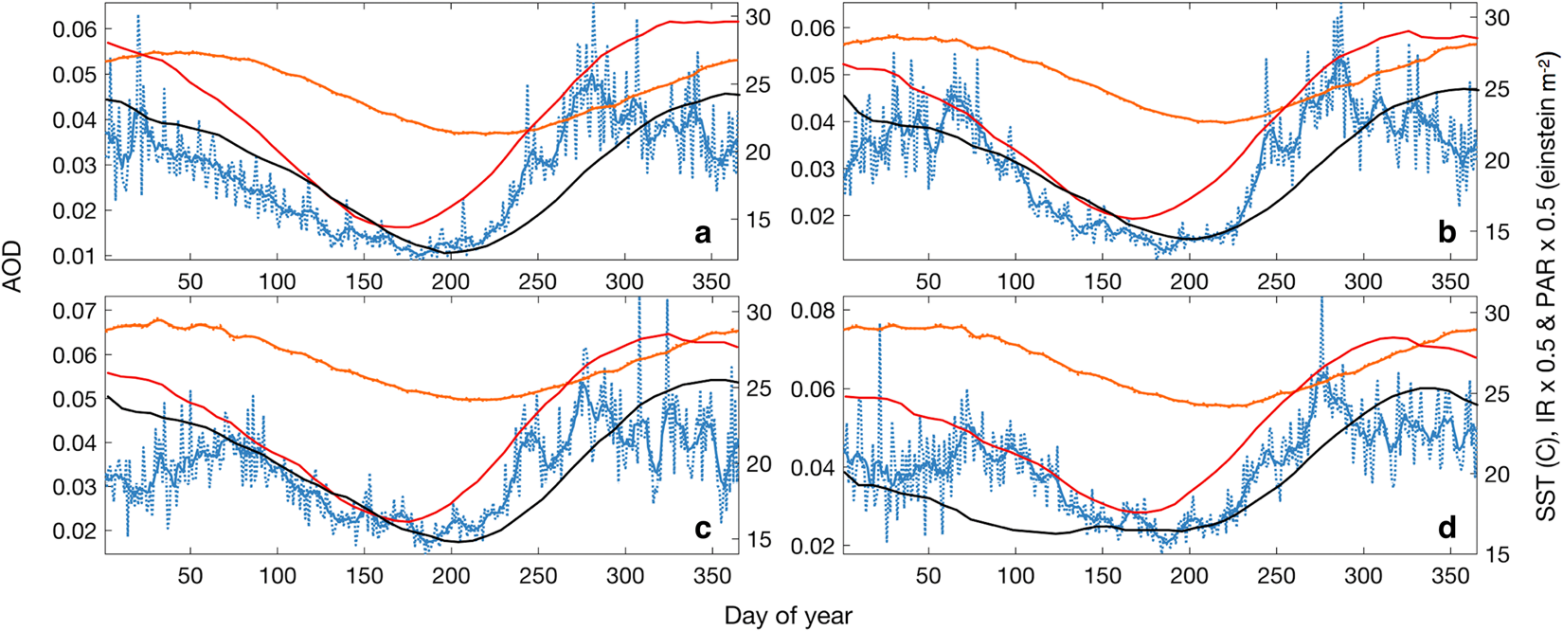
Mean daily area-averaged aerosol optical depth (AOD: blue) and sea surface temperature (SST: orange) and 8-day photosynthetically available radiation (PAR: red) and coral irradiance stress (IR: black) (2000–2017) at the (**a**) southern, (**b**) central, (**c**) northern and (**d**) far northern Great Barrier Reef (GBR). Solid lines represent the 8-day moving average for daily AOD and SST.

Figure 1 shows mean 8-day (computed for the period 2000 to 2017) AOD, SST, PAR and IR at the four study locations. AOD positively correlated with SST, PAR and IR at all sites (Table 1), with all four parameters displaying approximately synchronous seasonal variability. This relationship has been demonstrated between SST, irradiance and emissions of aerosol precursor compounds by marine biota in regions of the clean MBL previously^18,40^. The positive correlations reported here are therefore suggestive of a strong biogenic aerosol source over the GBR.

**Table 1:** Spearman’s ranked correlation coefficients (ρ) for 8-day data for 2000–2017. Values in bold are statistically significant (p < 0.001). See Supplementary Table S2 for all P values.

ρ	AOD	SST	IR	PAR	NT	WS	CHL	k490
**Southern GBR (** ***n*** ** = 690)**								
AOD	1.00	**0.39**	**0.67**	**0.70**	**0.38**	−0.05	**−0.24**	**−0.22**
SST		1.00	**0.30**	**0.39**	**0.37**	**0.21**	**0.17**	**0.26**
IR			1.00	**0.98**	**0.28**	**−0.17**	**−0.45**	**−0.43**
PAR				1.00	**0.42**	**−0.13**	**−0.35**	**−0.31**
NT					1.00	0.04	−0.12	−0.07
WS						1.00	**0.17**	**0.19**
CHL							1.00	**0.90**
k490								1.00
**Central GBR (** ***n*** ** = 682)**								
AOD	1.00	**0.49**	**0.57**	**0.61**	**0.48**	−0.08	−0.09	−0.02
SST		1.00	**0.31**	**0.42**	**0.49**	−0.12	**0.33**	**0.47**
IR			1.00	**0.98**	**0.41**	**−0.34**	**−0.45**	**−0.40**
PAR				1.00	**0.54**	**−0.32**	**−0.36**	**−0.29**
NT					1.00	**−0.15**	**−0.13**	−0.05
WS						1.00	**0.29**	**0.27**
CHL							1.00	**0.91**
k490								1.00
**Northern GBR (** ***n*** ** = 667)**								
AOD	1.00	**0.35**	**0.37**	**0.41**	**0.32**	**−0.16**	−0.09	0.01
SST		1.00	**0.27**	**0.38**	**0.57**	**−0.40**	0.04	**0.22**
IR			1.00	**0.98**	**0.27**	**−0.47**	**−0.46**	**−0.40**
PAR				1.00	**0.40**	**−0.49**	**−0.38**	**−0.29**
NT					1.00	**−0.30**	−0.06	0.06
WS						1.00	**0.26**	**0.19**
CHL							1.00	**0.83**
k490								1.00
**Far northern GBR (** ***n*** ** = 658)**								
AOD	1.00	**0.40**	**0.31**	**0.48**	**0.42**	**−0.27**	**−0.19**	−0.09
SST		1.00	**−0.13**	**0.32**	**0.85**	**−0.62**	−0.03	0.11
IR			1.00	**0.84**	−0.1	**−0.14**	**−0.51**	**−0.51**
PAR				1.00	**0.38**	**−0.42**	**−0.43**	**−0.34**
NT					1.00	**−0.58**	−0.11	0.01
WS						1.00	**0.15**	0.06
CHL							1.00	**0.79**
k490								1.00

**Figure 2:**
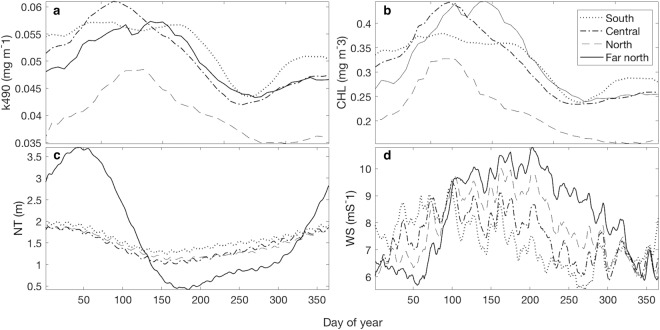
Mean 8-day (**a**) diffuse attenuation coefficient (k490), (**b**) chlorophyll-a (CHL), (**c**) noon tide height (NT) and (**d**) wind speed (WS) for the period 2000–2017.

As expected, IR was mainly predicted by PAR and was greatest when water turbidity, as measured by k490, was lowest (Fig. 2a). CHL (Fig. 2b) was the primary determinant of k490 at all sites, consistent with the findings of Cropp *et al*. (2018), and therefore negatively correlated with the irradiance metric (Table 1). IR positively correlated with SST and NT at the southern, central and northern GBR, due to seasonal increases in PAR, temperature and tide height in summer as expected. Conversely, IR negatively correlated with SST and NT at the far northern GBR site. The range of midday tide heights was high at this site (Fig. 2c), where extremely low winter and spring midday tides (<1 m) affected the irradiance metric and consequently reduced the dependence of IR on PAR alone (Table 1). The results suggest that aerosol concentration over the GBR is strongly influenced by irradiance levels, which are compounded by high SST in spring and summer and extremely low tides in winter. These are conditions during which corals enhance emissions of aerosol precursor compounds^15,36,37^, suggesting that the 2,300 km stretch of coral reefs are a substantial source of biogenic aerosol.

Conversely, AOD did not positively correlate with CHL at any site (Table 1), suggesting that phytoplankton are not major contributors to the aerosol burden over the GBR. This is supported by Fig. 2b which shows that peak CHL and therefore phytoplankton biomass, occurred in late summer after the peak in AOD. Wind direction and strength may also affect AOD, through competing influences that transport air masses to or away from the source location^50,51^ and the production of sea-spray via wind-driven mechanical disruption of the ocean surface and bubble-bursting processes^52,53^. AOD did not correlate with WS in the southern half of the GBR, yet weakly negatively correlated at the two northern sites (Table 1), reflecting aerosol advection out of the study region with high wind speeds and conversely, enhanced local emissions during calm conditions^8^. This is supported by seasonal variation in the WS climatology (Fig. 2d), whereby periods of low daily mean WS coincide with peak AOD.

**Figure 3:**
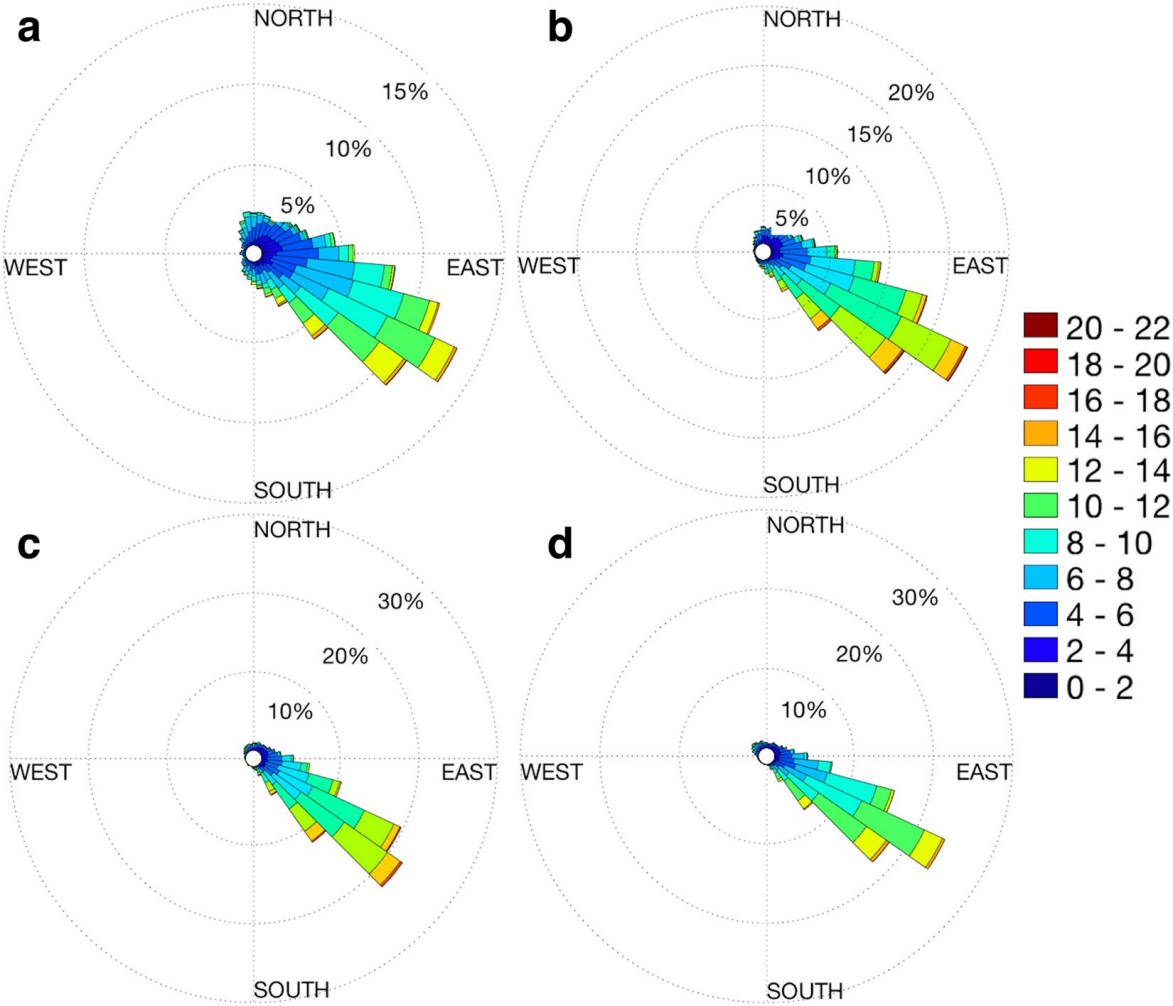
Wind rose of mean daily wind speed (WS) and direction (blowing from) at 10 m above sea-level at the (**a**) southern, (**b**) central, (**c**) northern and (**d**) far northern Great Barrier Reef (GBR). Bars represent the percentage of days that each wind direction occurred, with coloured portions indicating mean speed (ms^−1^). Calm conditions (WS < 2 ms^−1^) occurred on approximately 1% of days.

Although the transport of mineral dust from arid regions of Australia may influence AOD, the lack of positive correlation between AOD and WS and the dominance of easterly winds (Fig. 3) suggests otherwise. Previous modelled simulations of dust transport and wind direction have shown that while dust-storm activity is greatest during late spring in north-eastern Australia, the total influence of dust and continental particles on aerosol over the GBR is low^8,54^. Although it is not possible to identify the origin of aerosol particles using remotely sensed data alone, the results presented here are suggestive of a local biogenic aerosol source over the GBR.

**Figure 4:**
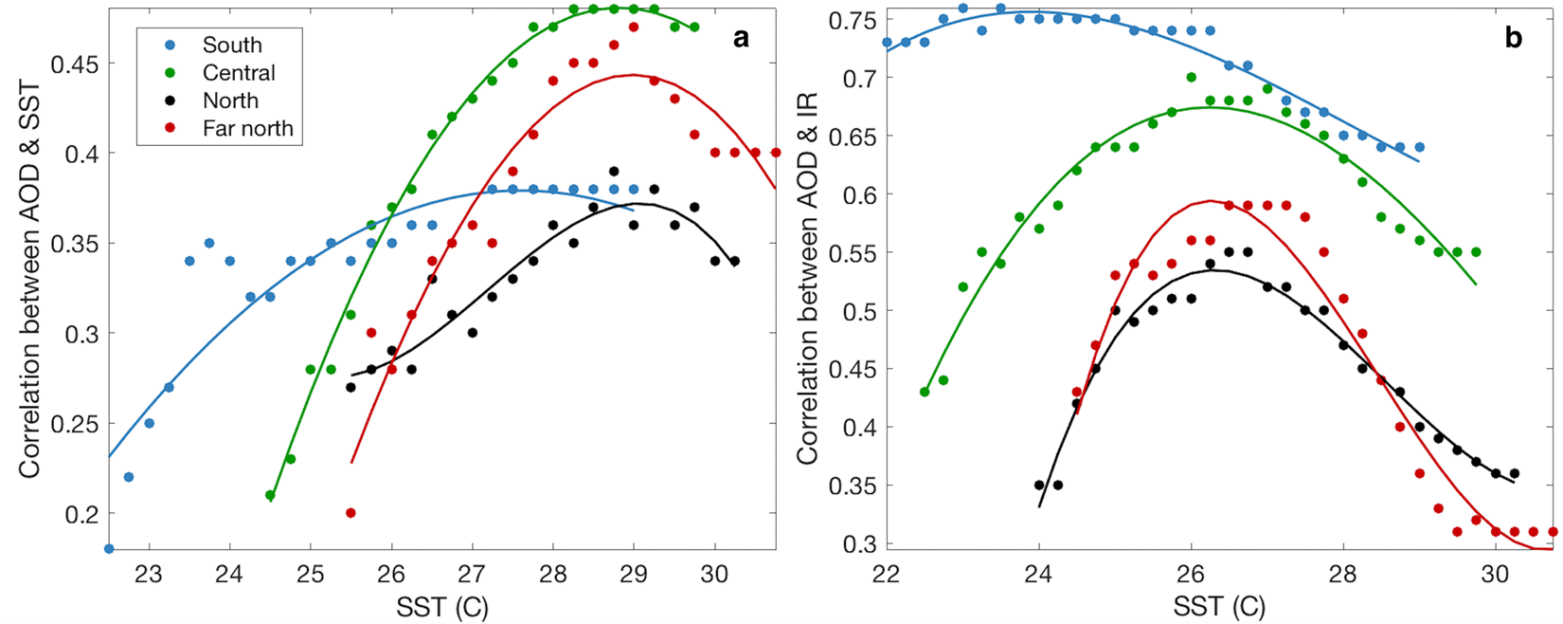
Correlation of (**a**) aerosol optical depth (AOD) and sea surface temperature (SST) and (**b**) AOD and coral irradiance stress (IR), as a function of SST. Each point represents the correlation for days when SST was equal to or less than the corresponding x-axis temperature. Thus, values of ρ at the maximum SST for each site represent the correlation between AOD and either SST or IR for all available days. Only statistically significant (p < 0.001) correlations are included.

Interestingly, summer SST and PAR coincided with high frequency variability in AOD (Fig. 1). Furthermore, correlations between AOD and both SST and IR approached zero for days where SST exceeded the MMM. This point occurred in February at all sites and ranged from 27.3 °C at the southern GBR, 28.5 °C at the central GBR and 29.1 °C at both northern sites. Figure 4a shows that the correlation between AOD and SST improved until SST approached the MMM for each site. The lack of improvement in AOD and SST correlation at this point is reflective of the negative or zero Spearman’s rank correlation coefficients (ρ) for SST beyond the MMM. Similarly, Fig. 4b shows that the correlation between AOD and IR improved with SST until temperatures reached approximately 1 °C below the MMM. At this point the correlation strength declined. Again, this suggests that aerosol emissions over the GBR are strongly influenced by the combined effects of high light levels and temperature and that a tipping-point in the relationship between biogenic aerosol emissions and coral thermal stress exists.

A similar pattern is observed in the limited field data available, where atmospheric DMS concentration is negatively correlated with SST during February 2016 (ρ = −0.35, p < 0.001, *n* = 274). SST exceeded 27.3 C (MMM for this site) on all but one day for the duration of the field measurements. Given that corals are considered to be stressed when SST exceeds the MMM, a negative correlation was expected between DMS and SST. Similarly to the correlation between AOD and SST, a peak positive correlation occurred between DMS and SST at 27 °C (ρ = 0.22, p < 0.05, n = 163), although correlation strength was weak likely due to persistently high SST. Correlation strength then approached zero for SST > 27.3 °C and became negative for SST > 28 °C.

### Mass coral bleaching events

Seven mass coral bleaching events have affected the GBR over the past two decades. These occurred due to extreme SST in the summers of 1998, 2002, 2006, 2016 and most recently in 2017^1,55,56^. Localised, yet widespread coral bleaching and mortality also occurred in the summers of 2009 and 2011 due to the combined effects of elevated SST, tropical storms and extreme rainfall^57,58^. As the correlation between AOD and SST over the 18-year study period approached zero for temperatures greater than the coral thermal stress threshold, anomalies for both parameters were examined before, during and after four mass coral bleaching events that occurred primarily due to extreme SST (2002, 2006, 2016 and 2017). The 1998 event was not examined as satellite data was only available for 2000 onwards. Given the large spatial extent of each site, periods of coral bleaching are coarsely defined based on the presence and severity of bleached coral in annual coral reef surveys conducted by AIMS and the Great Barrier Reef Marine Park Authority (GBRMPA).

#### Year 2002

**Figure 5:**
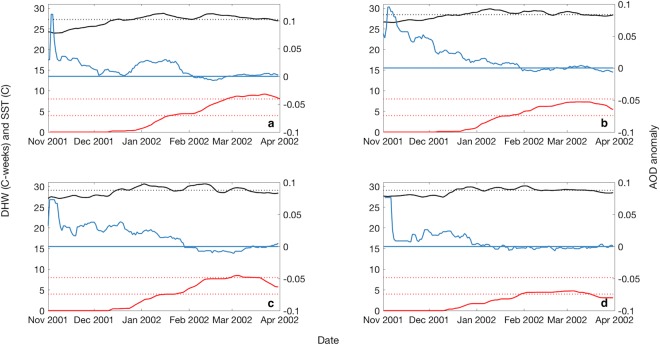
Moving average of 30-day aerosol optical depth (AOD) anomaly (blue), 8-day sea surface temperature (SST: black solid line) and degree heating week (DHW: red solid line) during the 2002 mass coral bleaching event at the (**a**) southern, (**b**) central, (**c**) northern and (**d**) far northern Great Barrier Reef (GBR). AOD was averaged over a moving 30-day window to smooth the data for visualization. Black dotted line represents the climatological mean monthly maximum (MMM). Red dotted lines represent the 4 °C-weeks and 8 °C-weeks critical coral bleaching thresholds.

Widespread coral bleaching occurred throughout the GBR from January to March 2002, with 41% of offshore and 72% of inshore reefs displaying moderate to high levels of coral bleaching^1^. Recovery was generally rapid, with the exception of reefs near Bowen in the central GBR which suffered up to 70% coral mortality. AOD was variable and often above the long-term average (LTA) for 2000–2017 from November to January (Fig. 5), suggestive of elevated biogenic aerosol emissions in response to rising temperatures. When SST exceeded the MMM and DHW approached or exceeded the coral bleaching critical threshold of 4 °C-weeks in January (Fig. 5), AOD declined to normal or below average levels for the remainder of the summer.

#### Year 2006

**Figure 6:**
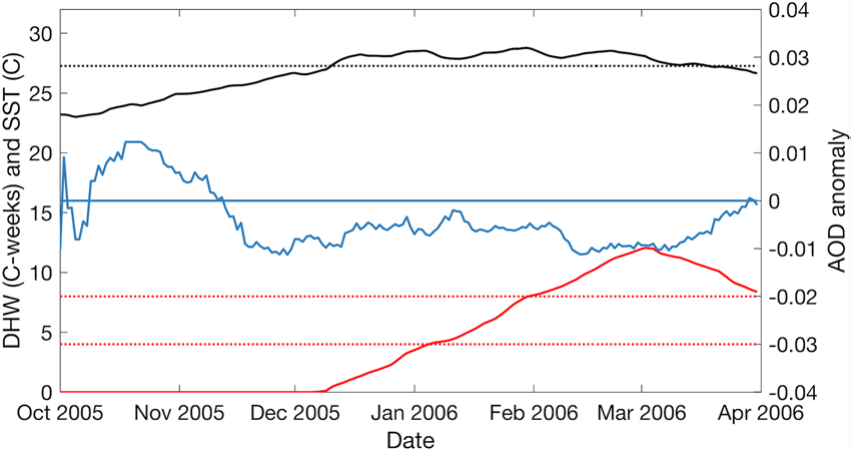
Moving average of 30-day aerosol optical depth (AOD) anomaly (blue), 8-day sea surface temperature (SST: black solid line) and degree heating week (DHW: red solid line) during the 2006 mass coral bleaching event at the southern Great Barrier Reef (GBR). Black dotted line represents the climatological mean monthly maximum (MMM). Red dotted lines represent the 4 °C-weeks and 8 °C-weeks critical coral bleaching thresholds.

Extreme SST in the southern GBR resulted in localised, moderate to severe coral bleaching throughout the Capricorn Bunker and Keppel Island regions respectively, from January to late March 2006^55^ and coincided with accumulated thermal stress well above 4 °C-weeks (Fig. 6). In October 2005, SST and AOD increased simultaneously until SST approached the MMM in November and continued to rise as AOD declined and remained exclusively below average until late March when accumulated thermal stress began to subside (Fig. 6).

#### Years 2016 and 2017

**Figure 7:**
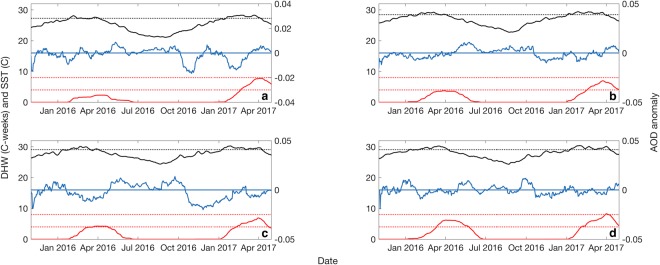
Moving average of 30-day aerosol optical depth (AOD) anomaly (blue), 8-day sea surface temperature (SST: black solid line) and degree heating week (DHW: red solid line) during the 2016 and 2017 mass coral bleaching events at the (**a**) southern, (**b**) central, (**c**) northern and (**d**) far northern Great Barrier Reef (GBR). Black dotted line represents the climatological mean monthly maximum (MMM). Red dotted lines represent the 4 °C-weeks and 8 °C-weeks critical coral bleaching thresholds.

During the summer of 2016, extreme SST accompanied by a strong El Niño system triggered one of the worst coral bleaching events on record, with 93% of reefs in the GBRMP affected, including a loss of two-thirds of coral in the northern half of the GBR and 29% of shallow water corals reef-wide^56^. Coral bleaching was most severe in the northern half of the GBR during the 2016 summer and to a lesser extent the central GBR^59^, with accumulated thermal stress meeting or exceeding the critical 4 °C-weeks threshold at each of these sites (Fig. 7b–d). Corals in the southern GBR were largely unaffected by this event as SST rapidly subsided with category 5 tropical cyclone Winston in February. SST remained above the long-term winter average in 2016 and by the following summer, accumulated thermal stress resulted in a second, consecutive bleaching event. The region of severe coral bleaching shifted further south in 2017, with the worst affected reefs in the northern and central GBR^60^. DHW approached the critical mass coral bleaching and mortality threshold of 8 °C-weeks at all sites during this event. AOD was highly variable and often below the LTA over the 1.5-year period (Fig. 7). Negative AOD anomalies are more frequent and often greater in magnitude than positive anomalies during both summer periods, particularly in the northern GBR which was worst affected by both bleaching events (Fig. 7c).

## Discussion

In the relatively unpolluted MBL of the southern hemisphere, biogenic sulfate emissions constitute a major source of fine-mode aerosol^12,13,61,62^. Corals and their endosymbionts are among the greatest sources of this sulfate through emissions of DMS and other volatile organic compounds (VOCs), particularly when corals experience physiological stress from high SST, irradiance and aerial exposure at low tide^15,16,36,63,64^. Phytoplankton and non-biogenic sources such as sea-salt spray and organic matter also contribute to the fine-mode aerosol burden^19,52,53,62^ however, analysis of phytoplankton biomass, WS and the likelihood of continental dust loading^54^ throughout the 18-year study period were not coherent with AOD.

It is hypothesized that enhanced DMS emissions increase the formation of MBA, which may form CCN and affect cloud microphysics and cover, thus acting as a biologically-derived negative feedback on SST^11^. Positive correlations have been found between DMS and SST and between DMS and irradiance in the GBR previously^36,40^, reflecting enhanced coral DMS emissions during thermal stress. The positive correlation between AOD and both SST and irradiance presented here may therefore reflect enhanced DMS-derived particle formation over the GBR. Importantly however, the correlation between AOD and SST approached zero for SST above the relative MMM for each site^46,47^. A tipping point in the correlation between AOD and coral irradiance stress was also apparent, whereby aerosol concentration increased with IR until SST reached approximately 1 °C below the MMM, at which point the correlation strength declined.

A wide range of evidence suggests that DMSP plays an essential role in the coral antioxidant response, whereby increasing temperature, light or aerial exposure with low tide, upregulates the biosynthesis of DMSP^20,21,40^. When the rate of DMSP production and breakdown to DMS is greater than conversion to dimethyl sulfoxide (DMSO) by ROS, a portion of DMS is emitted to the atmosphere. However, when the rate of consumption exceeds that of production, DMS emissions have been shown to decline. In chamber experiments, DMS emissions are reduced by up to 92% when *Acropora* sp. are exposed to elevated temperatures^9,21^. The decline in correlation strength between AOD and both SST and IR observed here may therefore reflect a decline in DMS-source strength with an upregulation of the coral antioxidant response. The correlation between SST and field DMS data at the southern GBR followed a similar trend to that of satellite-derived SST and AOD, with a peak positive correlation at approximately 27.3 °C, beyond which correlation strength approached zero or became negative. This provides further evidence of a strong coralline aerosol source over the southern GBR and supports our theory of a tipping-point in the coral stress-response.

This may explain why declines to normal or negative AOD anomalies occurred prior to and during four mass coral bleaching events. It is acknowledged that AOD over the GBR is likely not exclusively of biogenic origin, with sea-spray and other non-biogenic particles likely influencing the observed high variability. However, negative AOD anomalies corresponded to SST greater than coral thermal stress thresholds and may reflect a reduction in aerosol precursor emissions as corals experienced extreme stress events, thus providing further evidence that corals in the GBR are a source of MBA above background oceanic levels^8,15^. Interestingly, SST at the southern GBR was above the MMM for more than two months during the 2002 bleaching event and more than three months during both 2006 and 2017 events, yet for only around one month during the summer of 2016 when bleaching did not occur at this site. This suggests that corals in the southern GBR possess a level of temperature tolerance where SST must remain elevated for longer periods of time to cause coral bleaching compared to corals in the northern GBR. Additionally, local-scale variation in cloud cover may have influenced spatial patterns of coral bleaching^65^, protecting corals in the far southern GBR during this event.

Although the extent to which coral reef DMS emissions contribute to the formation of new aerosol particles and affect climate is not certain, a reduction in DMS source strength with rising SST could lead to a decline in CCN formation and thus, low-level, high albedo cloud cover. Ultimately, this would increase the amount of solar radiation absorbed by the ocean, lead to further ocean warming and the establishment of a positive feedback on SST. This may be an additional reason why mass coral bleaching events are increasing in frequency and severity globally, particularly in those regions where coral health and cover is low due to poor water quality, disease and predators^3,4,66^. A reduction in coral reef-derived aerosol may also have adverse effects far from emission sources, where for example, corals in the southern GBR can act as a source of DMS and therefore secondary aerosol for the central and northern reef when south-east trade winds prevail^10,15,36^. A reduction in DMS emissions at a local scale could lead to a reduction in aerosol formation at reefs downwind of the source location, potentially exacerbating coral stress and bleaching.

Given it’s southern hemisphere location, large spatial extent and the dominance of easterly trade winds, the GBR provides a unique and valuable study location for remote sensing analysis of reefal aerosols. In contrast, analysis of MBA from satellite-derived AOD is limited many other regions of the world, due to confounding loads of anthropogenic aerosol and other natural particles, particularly in the northern hemisphere. Although there are several other coral reef systems which could provide a valuable study location (e.g. French Polynesia) these are often largely composed of continental reefs and small, scattered atolls. Analysis of the effects of ocean warming and coral bleaching on AOD in these regions is an important area for future research however, will require higher resolution data.

The continued degradation of coral reefs globally has led to the proposal of several strategies to mitigate the warming effects of GHG. One promising strategy involves artificially injecting sea-spray or sulfate particles into the atmosphere to essentially mimic biogenic aerosols and increase local ocean albedo over coral reefs. In a modelled scenario, injecting 5 Tg SO_2_ annually into the atmosphere above coral reefs in the Caribbean reduced SST, irradiance and sea-level rise and resulted in almost no predicted coral bleaching events over the next 50 years^67^. This approach would be expensive and inefficient over the long-term for large coral reef systems such as the GBR, however will likely be necessary to protect vulnerable or high value coral reefs in future with ongoing ocean warming. Future research is needed to accurately quantify and characterise coral reef-derived aerosol emissions at both an ecosystem and species level. Doing so would provide valuable insight into how to enhance corals natural protective abilities, thus reducing the dependence of coral conservation on costly artificial mitigation strategies.

## Conclusion

The results presented here suggest that corals in the GBR can act as a substantial source of biogenic aerosol and support recent findings that corals can influence local atmospheric properties through stress-induces emissions of DMS and other VOCs^8,15,63^. Importantly however, if ocean warming exceeds the tipping point we identify, the ability of corals to influence their local environment may be impaired. Although the derivatives of DMSP are important sources of aerosol precursor compounds, DMSP is also an important compound in the coral antioxidant response^20,21,68^. The level of coral stress and thus, the consumption rate of DMSP, is therefore an important determinant of coralline aerosol precursor emissions.

We hypothesize that the reduction in positive correlations between AOD and SST and between AOD and IR for temperatures above coral thermal stress thresholds, reflects a reduction in aerosol precursor emissions. From these findings, we posit that corals exhibit a two-stage stress response, whereby increases in temperature increase aerosol precursor emissions, potentially reducing coral stress through changes to local atmospheric properties. However, when SST exceeds coral physiological limits, aerosol precursor emissions shut-down as corals attempt to cope with oxidative stress. Although a biological ocean “thermostat” may exist within the GBR, rising ocean temperatures are likely weakening this mechanism and may explain why the incidence and severity of mass coral bleaching events is on the rise. Conserving the world’s coral reefs is an important issue and regardless of the approach, there is substantial incentive to increase our understanding of natural aerosol processes. This information is vital for the future protection and management of coral reefs worldwide and is of the utmost importance to coral reef managers such as the GBRMPA, industry such as tourism, fisheries and agriculture, and climate researchers.

## Electronic supplementary material

Supplementary Information

## Data Availability

The datasets analysed during the current study are available from the corresponding author on request.
