# Supplementary material for: Effects of ocean warming and coral bleaching on aerosol emissions in the Great Barrier Reef, Australia
Source: Sci Rep. 2018 Sep 19;8:14048. doi: 10.1038/s41598-018-32470-7 (PMC6145874; doi:10.1038/s41598-018-32470-7)
Supplement: Supplementary file 1 — Supplementary Information [file 41598_2018_32470_MOESM1_ESM.pdf]

Effects of ocean warming and coral bleaching on aerosol emissions in the Great Barrier Reef, Australia

Rebecca Jackson[1, 2], Albert Gabric[2, 3] & Roger Cropp[1]

[1] School of Environment and Science, Griffith University, Gold Coast, 4222, Australia

[2] Australian Rivers Institute, Griffith University, Gold Coast, 4222, Australia

[3] School of Environment and Science, Griffith University, Nathan, 4111, Australia

Supplementary Figure S1. Great Barrier Reef Marine Park (GBRMP) management zones showing the location of sampling grids used in the analysis.

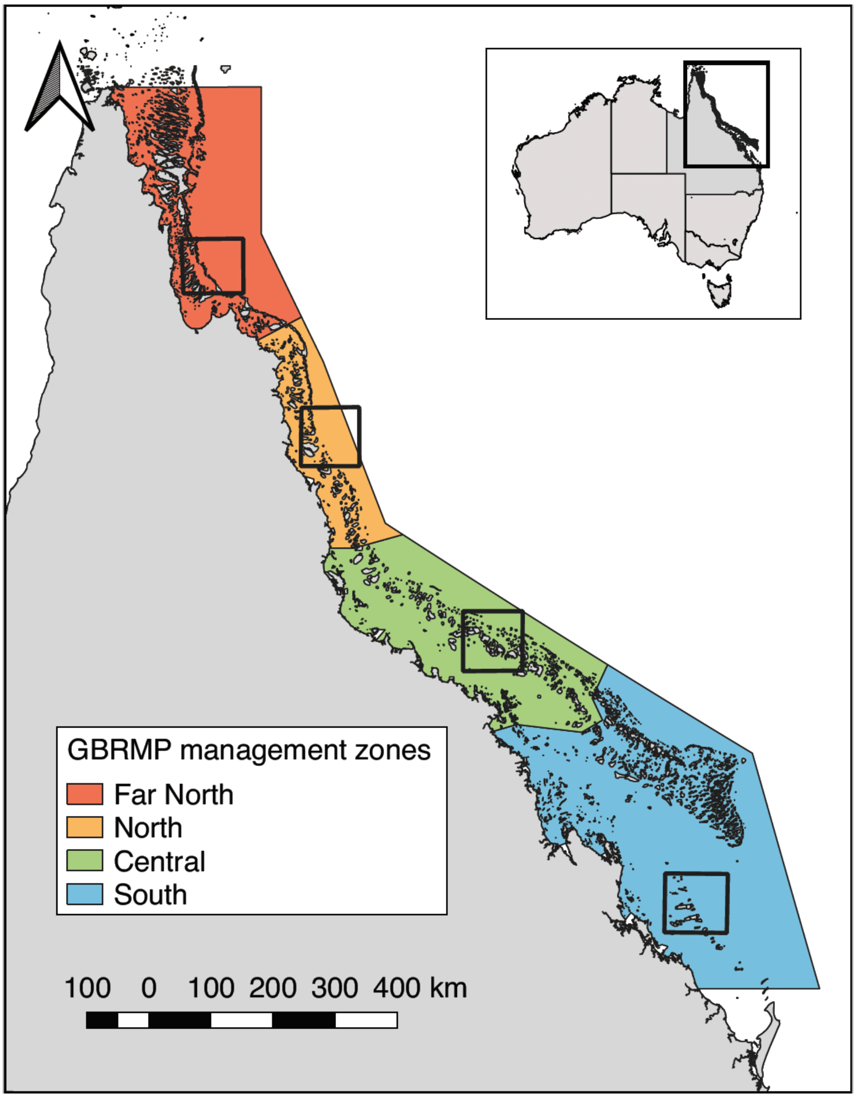

Supplementary Table S2. Significance (*p*) values for Spearman's ranked correlation coefficients for 8-day data for 2000-2017 (α=0.001).

p	AOD	SST	PAR	IR	NT	WS	CHL	k490
Southern GBR (*n*=690)								
AOD	1.000	0.000	0.000	0.000	0.000	0.167	0.000	0.000
SST		1.000	0.000	0.000	0.000	0.000	0.000	0.000
PAR			1.000	0.000	0.000	0.000	0.000	0.000
IR				1.000	0.000	0.000	0.000	0.000
NT					1.000	0.306	0.001	0.052
WS						1.000	0.000	0.000
CHL							1.000	0.000
k490								1.000
Central GBR (*n*=682)								
AOD	1.000	0.000	0.000	0.000	0.000	0.044	0.021	0.590
SST		1.000	0.000	0.000	0.000	0.001	0.000	0.000
PAR			1.000	0.000	0.000	0.000	0.000	0.000
IR				1.000	0.000	0.000	0.000	0.000
NT					1.000	0.000	0.001	0.198
WS						1.000	0.000	0.000
CHL							1.000	0.000
k490								1.000
Northern GBR (*n*=667)								
AOD	1.000	0.000	0.000	0.000	0.000	0.000	0.022	0.758
SST		1.000	0.000	0.000	0.000	0.000	0.350	0.000
PAR			1.000	0.000	0.000	0.000	0.000	0.000
IR				1.000	0.000	0.000	0.000	0.000
NT					1.000	0.000	0.123	0.152
WS						1.000	0.000	0.000
CHL							1.000	0.000
k490								1.000
Far northern GBR (*n*=658)								
AOD	1.000	0.000	0.000	0.000	0.000	0.000	0.000	0.027
SST		1.000	0.000	0.0005	0.000	0.000	0.419	0.004
PAR			1.000	0.000	0.000	0.000	0.000	0.000
IR				1.000	0.010	0.000	0.000	0.000
NT					1.000	0.000	0.003	0.847
WS						1.000	0.000	0.117
CHL							1.000	0.000
k490								1.000
